# The Glu143 Residue Might Play a Significant Role in T20 Peptide Binding to HIV-1 Receptor gp41: An In Silico Study

**DOI:** 10.3390/molecules27123936

**Published:** 2022-06-20

**Authors:** Ahmed L. Alaofi

**Affiliations:** Department of Pharmaceutics, College of Pharmacy, King Saud University, Riyadh 11451, Saudi Arabia; ahmedofi@ksu.edu.sa

**Keywords:** enfuvirtide, gp41, HIV-1 receptor, molecular dynamics simulations, principal component analysis, free energy landscape

## Abstract

Despite the enormous efforts made to develop other fusion inhibitors for HIV, the enfuvirtide (known as T20) peptide is the only approved HIV-1 inhibitory drug so far. Investigating the role of potential residues of the T20 peptide’s conformational dynamics could help us to understand the role of potential residues of the T20 peptide. We investigated T20 peptide conformation and binding interactions with the HIV-1 receptor (i.e., gp41) using MD simulations and docking techniques, respectively. Although the mutation of E143 into alanine decreased the flexibility of the E143A mutant, the conformational compactness of the mutant was increased. This suggests a potential role of E143 in the T20 peptide’s conformation. Interestingly, the free energy landscape showed a significant change in the wild-type T20 minimum, as the E143A mutant produced two observed minima. Finally, the docking results of T20 to the gp41 receptor showed a different binding interaction in comparison to the E143A mutant. This suggests that E143 residue can influence the binding interaction with the gp41 receptor. Overall, the E143 residue showed a significant role in conformation and binding to the HIV-1 receptor. These findings can be helpful in optimizing and developing HIV-1 inhibitor peptides.

## 1. Introduction

The human immunodeficiency virus (HIV) is a causative agent for acquired immunodeficiency syndrome (AIDS) infection, a chronic and life-threatening disease. HIV utilizes its envelope glycoproteins to initiate the entry process into host cells [1]. Specifically, transmembrane glycoprotein 41 (gp41) and surface glycoprotein 120 (gp120) of HIV mediate viral entry into host cell membranes. The binding of gp120 to cell receptor CD4+ and a chemokine receptor (CCR5 or CXCR4) promotes a series of conformational changes that allows insertion of the fusion protein (FP), located at the N-terminus of gp41, into the cell membrane [2,3].

The enfuvirtide (T20) peptide was the first member of HIV-1 entry inhibitors granted FDA approval for clinical use to treat HIV infection, which occurred in 2003 [4,5]. The T20 peptide has 36 amino acids that originally derive from gp41. The T20 peptide prevents the required conformational changes of gp41 that are essential for HIV-1′s fusion to host cell receptors [6]. Although the exact functional mechanism of the T20 peptide’s action has not been solved yet, binding studies show that the T20 peptide interact with several regions in gp41. These gp41 regions are the N-terminal heptad repeat (NHR), C-terminal heptad (CHR) helices, the fusion peptide (FP) as well as the transmembrane domain (TDM) [4]. Therefore, the T20 peptide’s interaction with gp41 inhibits the necessary conformational changes in gp41 and eventually blocks HIV from binding to host cell receptors.

Despite the enormous efforts to develop other fusion inhibitors for HIV, the T20 peptide is the only approved HIV-1 inhibitory drug so far. The challenges of developing new HIV inhibitors are attributed to several factors such as the high mutation rate of HIV envelope proteins (i.e., sequence variability) and lack of knowledge regarding the exact mechanism of action of the T20 peptide. Moreover, the glycosylation pattern of gp41 envelope proteins act as a “glycan shield” for the conserved (i.e., non-mutated) regions [1].

Molecular dynamics (MD) simulations is a powerful tool for studying biomolecular structures as well as identifying conformational-dependent residues in proteins [7]. Moreover, integrating MD simulations with docking techniques might provide complementary information, as there resulting trajectories are used in docking studies [8]. Therefore, investigating the role of potential residues in the T20 peptide can be helpful for understanding the role of these residues and can help in optimizing and developing new HIV-1 inhibitory drugs. However, in the literature, there is little investigation regarding conformational-dependent residues for inhibitory peptides such as the T20 peptide. Therefore, we studied the wild-type (WT) T20 peptide along with its E143A mutant using MD simulations and docking techniques. The E143 residue is one of the few T20 residues that interacted with multiple gp41 residues.

## 2. Results and Discussion

### 2.1. RMSD and RMSF Assessments

**Figure 1:**
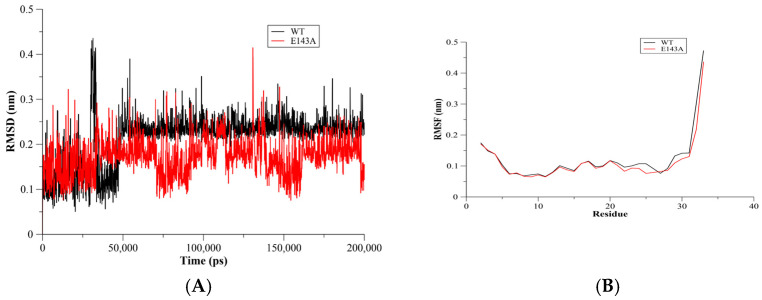
C-α root mean square of deviation (RMSD) for the WT Env (red) and the E143A (blue) mutant (**A**) The convergence of both systems was established as indicating stable MD simulation systems. For the C-α root mean square of fluctuation (RMSF), the E143A mutant showed slightly rigid residues during the MD simulations than WT T20 (**B**).

The C-α root mean square deviation (RMSD) method was used to assess the stability of the MD simulation systems. The WT T20 and E143A mutant systems showed a plateau convergence after 50 ns of the MD simulations’ run (Figure 1A). This indicated stable systems for the WT and its E143A mutant during the MD simulations. Moreover, the simulation results showed that the RMSD values of both the WT T20 and E143A systems were similar at most time points during the simulations. The fluctuation assessment of WT T20 and the E143A mutant during the MD simulations were conducted using C-α root mean square flexibility (RMSF) analysis. It was anticipated that in short peptides, flexibility might not be observed due to the short sequences. However, in a short sequence, the RMSF analysis showed that the E143A mutant had a slightly rigid structure in comparison to the WT T20 peptide (Figure 1B). The slightly flexible residues of WT T20 were in the E146-L150 region (Figure 1B). Identifying critical residues can be useful in understanding protein binding [9]. Our results showed that the flexibility of the T20 peptide was reduced slightly by removing the E143 residue. This suggests that the E143 residue might be a critical residue in T20 peptide conformation and binding, even though a single α-helix structure might not have a comparable motion of domains.

### 2.2. Conformational Changes

**Figure 2:**
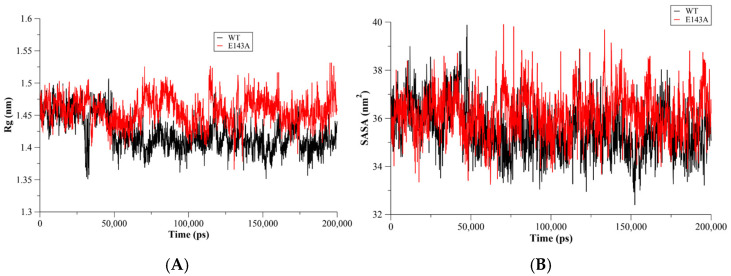
The radius of gyration (Rg) as a function of the MD simulation time (**A**). The WT Env (red) showed a compact conformation in comparison to the E143A mutant (blue). This might indicate the role of E18 in the Env’s compact conformation. The surface area solvent accessibility for the WT Env (blue) and its E18A mutant as a function of simulation time (**B**). There was no difference between the WT and its E18A mutant.

The radius of gyration (Rg) was used to assess the conformational compactness and the size of the proteins [10]. Therefore, the Rg of WT T20 and the E143A mutant were calculated during the MD simulations to monitor their conformational compactness during the simulations. The Rg of the all-protein atoms showed a more compact conformation for WT T20 in comparison to its E143A mutant. The Rg of T20 was enhanced due to the mutation, indicating the role of E143 in the conformational compactness of the T20 peptide. The E143A mutant showed a slight rigid structure but a loose conformation in comparison to WT T20 (Figure 2A). This might suggest the requirement of a certain degree of flexibility to maintain T20′s conformation for binding to the gp41 receptor. Moreover, the solvent accessible surface area (SASA) was used to predict conformational changes in WT and mutant proteins [11,12,13]. There was a slight enhancement in the SASA of the E143A mutant compared to WT T20 (Figure 2B).

### 2.3. Principal Component Analysis (PCA)

**Figure 3:**
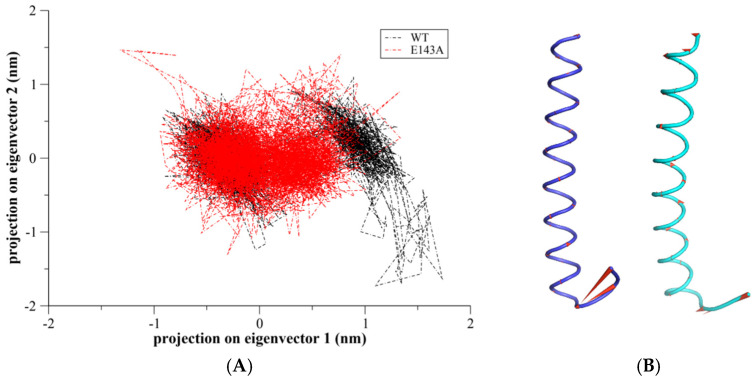
Projection of the motion of WT T20 (black color) aligned with the E143A mutant (red color) along with the first two principal eigenvectors (i.e., PC1 and PC2) in nm (**A**). Porcupine plots are depicted to show the motion across the first principal component (PC) in WT T20 (blue color) and E143A mutant (cyan color) peptides (**B**). The arrows reflect the direction of the correlated motion and the extent of the motion.

Principal component analysis (PCA) was used to identify major contributing components to the protein dynamics [14,15]. It is known that protein dynamics are confined within the first and second principal component (PC) modes. Alignment and projection of 2D eigenvectors for WT T20 and the E143A mutant would be helpful to monitor the mutation’s effect on the dynamic motions of T20. The alignment of the principal components of WT T20 and the E143A mutant showed an overlap in the conformational subspace (Figure 3A). The E143A mutant was found to sample from a narrower conformational subspace in comparison to WT T20. Moreover, the porcupine plot of PC1 showed that the C-terminus of WT T20 and the E143A mutant confined most of the motion (Figure 3B). However, the E143 residue was not part of the C-terminus, as the E143A mutation could rigidify the T20′s structure. The distant effect of E143 on the flexibility of neighboring residues (i.e., E146-L150) suggests E143 might play a role in the overall flexibility of the T20 peptide.

### 2.4. The Free Energy Landscape (FEL)

**Figure 4:**
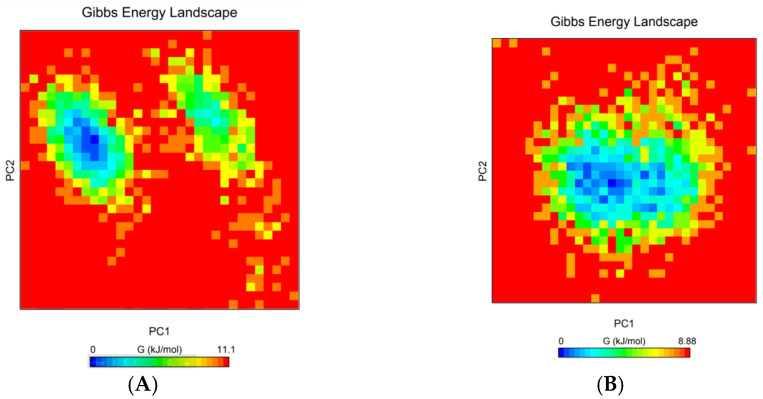
The free energy landscape (FEL) was plotted using PC1 and PC2 for the WT Env (**A**) and its E18A mutant (**B**) during the 100 ns MD simulations. The FEL showed that the A143 mutation changed the conformation of the T20 peptide.

The free energy landscape (FEL) of the all-peptide atoms was used to evaluate conformations at minima during the MD simulations [11]. The FEL of the backbone residues was plotted by projecting MD trajectories onto the first two principal components PC1 and PC2 of T20 and the E143A mutant. The WT T20 peptide showed two basins with a single minimum, while the E143A mutant showed several basins with minima (Figure 4). Mutation of E143 residue disrupted the single minimum in the E143A mutant. However, WT T20 showed only one dark-blue minimum, indicating one favorable conformation (Figure 4A). The E143A mutation might allow for sampling more than one conformation instead of only one conformation (Figure 4B). Overall, the E143 residue’s role in the T20 peptide might be important for the T20 peptide’s conformation and, therefore, in binding interaction to the gp41 receptor.

### 2.5. T20 Docking to the gp41 Receptor

**Figure 5:**
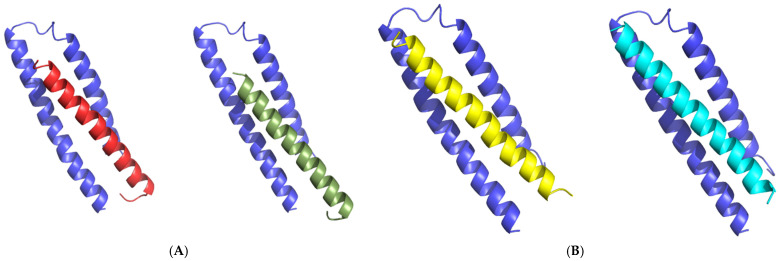
In (**A**), the T20_200ns_ frame structure was docked to gp41 receptor (blue color) using HADDOCK (T20 colored with red) and HawkDock (T20 colored with smudge). In (**B**), the E143A_200ns_ frame structure was docked gp41 receptor (blue color) using HADDOCK (E143A colored with yellow) and HawkDock (E143A colored with cyan).

Structural trajectories of WT T20 and the E143A mutant were utilized for docking with the gp41 receptor. The structures at 200 ns frames of WT T20 (T20_200ns_) and the E143A (E143A_200ns_) mutant were selected as a representative dynamic structure for each MD simulations system. Afterward, both the T20_200ns_ and E143A_200ns_ structures were docked to the X-ray structure of the gp41 receptor using multiple docking techniques HADDOCK and HawkDock servers. There was agreement in the docking results between both techniques especially in the first ranking models of HawkDock using MM-GBSA scoring (see Supplementary Materials Table S1). For E143A mutant, the first ranking model of HawkDock, model 1, was highly matched to of HADDOCK models. For WT T20, the second ranking model, model 4, showed similar docking of HADDOCK models. Therefore, matched models from both docking techniques were selected to compare the binding interaction for T20_200ns_–gp41 and E143A_200ns_–gp41 complexes (Figure 5). It was clear the E143A mutation changed the binding interaction of T20 peptide to the gp41 receptor. This suggests, the E143 residue might play a role in the binding of T20 peptide with its receptor.

## 3. Materials and Methods

### 3.1. Structural Preparation

The X-ray structure of the wild-type (WT) T20 peptide was obtained from the Protein Data Bank (PBD ID 5ZCX) The mutation of the T20 peptide was performed using PyMOL to obtain the E143A mutant [4]. The sequence of the WT T20 peptide was Ac-^127^YTSLIHSLIEE SQNQQEKNEQELLELDKWASL^158^, and the N-termini of both WT T20 and the E143E mutant were capped with an acetyl group. The obtained structure of the E143A mutant was validated as described previously [11,12].

### 3.2. MD Simulations

MD simulations for the WT T20 and E143A systems were performed using the GROMACS 5.1.4 program with the CHARMM27 force field [16]. The X-ray structure of WT T20 and the obtained E143A structure were used as a starting coordinate for the simulation systems. Afterward, MD simulations were conducted for both systems following our previous works with slight changes [11,12,17]. Particle mesh Ewald (PME) [18] was used instead of the cutoff scheme, and a short-range non-bonded cutoff distance of 1.3 nm was used to compute the long-range electrostatic interactions. Ionization states were assigned for titratable residues and the N- and C-termini according to a pH 7.0 condition. Each system was solvated with TIP3P water with a minimal distance of 0.407 nm between the solute and the wall of the cubic box of length 6.05711 nm [19]. The number of solvent and solute atoms were 546, 21208 and 541, 21210, for WY T20 and E143A systems respectively. Then, a proper number of ions (i.e., Na and Cl) were added instead of water molecules to simulate the 0.15 M ionic strength system. A brief energy minimization using the steepest descent algorithm was performed before a 100 ps long unconstrained equilibration MD simulation was conducted at a constant temperature (i.e., 300 K) and pressure using a Berendsen thermostat. Finally, a 200 ns long production MD simulation was conducted at a constant temperature of 300 K. The temperature was controlled using a velocity-rescale thermostat [18,20] and a time step of 2 fs.

### 3.3. Docking and Visualization

The online HADDOCK2.4 and HawkDock servers were used to dock the WT T20 peptide or the E143A mutant to their gp41 receptor (PDB ID 2OT5) [21,22]. For docking using HADDOCK, the active residues of T20 and gp41 were selected according to the binding interface in the T20–gp41 complex and surrounding regions. The T20 and E143A peptides’ active residues were located between L130 and W155, while the gp41′s active residues were I3-Q7, A13-Q17, G27-Q30, A33-S35, E47, I48, H56, S57, S62, and Q63. While for HawkDock server, T20 or E143A mutant were docked to gp41 receptor and reveal ten models. Afterward, MM-GBSA scoring was performed to rank the resulted models based on their binding affinity. Finally, first ranking models from HawkDock technique were selected with similar models from HADDOCK techniques. All peptide visualizations were performed using PyMOL 2.1 [23]. The RMSD, RMSF, Rg, and SASA were conducted using GROMACS 5.6 and the commands “gmx rmsf”, “gmx rmsd”, “gmx sasa”, “gmx gyrate”, and “gmx distance”, respectively.

## 4. Conclusions

The T20 peptide was the first member of the HIV-1 inhibitor class granted FDA approval. Investigating the potential residues in T20 might help us to overcome the current challenges faced in developing new inhibitor drugs for HIV-1. Our MD simulations study showed that the E143 residue of the T20 peptide played a significant role in the conformational structure of the peptide. The E143A mutation not only affected the conformations of the T20 peptide but also the binding interaction with the HIV-1 receptor (i.e., gp41). Although we conducted 200 ns long MD simulations, a longer simulation time might be more informative regarding peptide dynamics. Moreover, experimental studies might confirm these findings using structural instruments such as nuclear magnetic resonance (NMR) or surface plasmon resonance (SPR) studies.

## Data Availability

Data is contained within the article or Supplementary Materials.

## Supplementary Materials

The following supporting information can be downloaded at: https://www.mdpi.com/article/10.3390/molecules27123936/s1. Table S1: Ranking of docking models using MM-GBSA scoring.
