# Supplementary material for: The Glu143 Residue Might Play a Significant Role in T20 Peptide Binding to HIV-1 Receptor gp41: An In Silico Study
Source: Molecules. 2022 Jun 20;27(12):3936. doi: 10.3390/molecules27123936 (PMC9229102; doi:10.3390/molecules27123936)
Supplement: Supplementary file 1 [file molecules-27-03936-s001.zip › molecules-1584807-supplementary.pdf]

Article

The Glu143 residue might play a significant role in T20 peptide binding to HIV-1 receptor gp41: An In silico study

Ahmed L. Alaofi [1*]

Department of Pharmaceutics, College of Pharmacy, King Saud University, P.O. Box 2457, Riyadh 11451; ahmedofi@ksu.edu.sa

* Correspondence: ahmedofi@ksu.edu.sa

Table S1: Ranking of docking models using MM-GBSA scoring.

Ranking	WT T20	Binding free energy of complex (kcal/mol)
2	T20WT_model2	-42.13
4	T20WT_model4	-30.19
9	T20WT_model9	-28.83
3	T20WT_model3	-28.39
5	T20WT_model5	-27.13
8	T20WT_model8	-27.07
10	T20WT_model10	-26.94
7	T20WT_model7	-20.36
1	T20WT_model1	-14.43
6	T20WT_model6	-12.01

Ranking	E143A mutant	Binding free energy of complex (kcal/mol)
1	T20_mu_model1	-61.78
9	T20_mu_model9:	-55.38
6	T20_mu_model6	-43.45
5	T20_mu_model5	-42.82
2	T20_mu_model2:	-34.49
8	T20_mu_model8	-34.08
10	T20_mu_model10	-17.29
4	T20_mu_model4	-12.62
3	T20_mu_model3	-11.88
7	T20_mu_model7	-11.04
